# S6-2 Barriers and facilitators perceived by stakeholders regarding the implementation of physical activity interventions for people aged over 50

**DOI:** 10.1093/eurpub/ckad133.029

**Published:** 2023-09-11

**Authors:** Denise Peels, Janet Boekhout, Rieteke Hut

**Affiliations:** Open Universiteit, The Netherlands; Open Universiteit; Open Universiteit

## Abstract

**Aim:**

Although there are many proven effective physical activity (PA) interventions for middle-aged and older adults (MAOA), implementation in a real world setting is often lacking. Intermediary organizations (further referred to as ‘stakeholders’) often determine the final exposure of the intervention to the target population and therefore have a crucial role in the implementation process. This study provides insight in stakeholders’ perceptions of barriers and facilitators (i.e. implementation determinants) regarding implementation of PA interventions among MAOA.

**Method:**

A semi-structured interview guide was developed based on the Consolidated Framework for Implementation Research (CFIR), including five domains of implementation determinants, i.e.: interventions characteristics, outer setting (e.g., the target population’s needs and policies), inner setting (e.g., organizational goals ), individuals’ characteristics (e.g., stakeholders’ self-efficacy) and the implementation process. Using snowball sampling (starting with the regional health counselor) stakeholders were recruited to participate in the interviews. Thematic analyses, guided by a CFIR-based codebook, was applied.

**Results:**

We completed 25 interviews, involving representatives of regional health counselors (n = 5), municipalities (n = 3), sportservice organizations (n = 5), welfare organizations (n = 3), and several individual organizations. Analyses showed that in all CFIR domains relevant implementation determinants were identified. Stakeholders mentioned being able to play a relevant role in the implementation of PA intervention. Collaboration between organizations is essential to successfully perform all steps needed to implement interventions. Focusing on a broader perspective than increasing PA behavior, such as stimulating positive health seemed an important facilitator when implementing PA intervention. An often mentioned barrier was that most policy plans currently focus on youth, consequently resulting in a lack of means to implement interventions for MAOA.

**Conclusion:**

Gaining insight in the perception of stakeholders’ most relevant implementation determinants is a crucial step in the development of an effective implementation plan. In a next phase of our study, a collaboration meeting between stakeholders will be organized in which determinants will be ranked. The ranking of these determinants will be discussed with the symposium audience as well. Matching the implementation determinants, potential implementation strategies will be discussed.

